# Individually Delivered Parenting Program OPPI: Promising Results in Parental Perceptions and Children’s Behavioral Symptoms in Clinical Settings

**DOI:** 10.1177/13591045251407367

**Published:** 2025-12-19

**Authors:** Assi Peltonen, Vilja Seppälä, Heidi Backman, Taru Saarelainen, Tiia Kuha, Marjo Flykt, Eeva T. Aronen

**Affiliations:** 1 Children’s Hospital/Pediatric Research Center, Child Psychiatry, 3836 University of Helsinki and Helsinki University Hospital, Helsinki, Finland; 2 Welfare Sciences, Department of Psychology and Logopedics, Faculty of Social Sciences, Tampere University, Tampere, Finland; 3 Department of Psychology, Faculty of Medicine, 60655 University of Helsinki, Helsinki, Finland

**Keywords:** behavioral problems, child psychiatry, parenting program, individually delivered parenting program, disruptive disorders

## Abstract

Parenting programs are well-established treatments for children’s behavioral problems. However, engaging parents remains challenging, and a deeper understanding of how parents perceive these programs is needed to tailor them better respond to parents’ needs. This study introduces a novel individually delivered parenting program, OPPI. We investigate parental perceptions of the program’s content and teaching formats included and its preliminary effects on children’s behavioral symptoms. The participants were 61 parents with 45 children attending the intervention at the child psychiatric clinic at Helsinki University Hospital, Finland. Parents filled out questionnaires on family background, child’s symptoms, and their perceptions of the practices and teaching formats in the intervention. Parental overall opinion about the program was highly positive; especially the guidance given by counselors was considered of high quality. Practical teaching methods were perceived by parents as both the easiest and the most useful. Parents perceived most parenting practices as useful and easy to follow. Parents also reported that children’s behavioral symptoms decreased significantly from pre- to post-intervention. Our study provides valuable information on the feasibility of formats and contents in parenting program. This can guide clinicians in focusing treatment more effectively, ultimately enhancing parental engagement and the overall effectiveness of parenting programs.

## Plain Language Summary

Parenting programs help children with behavioral problems. However, it is hard to involve parents in these programs, and up to half of the children are left untreated. To get parents involved, we need to better understand their opinions of parenting programs. This study introduces a new individually delivered parenting program, OPPI, developed for families referred to hospitals for their child’s behavioral problems and evaluates parents’ opinions about the advantages and disadvantages of the program format and contents.

**Methods:**

Participants comprised 61 parents and their 45 children attending the intervention at the child psychiatric clinic in Helsinki University Hospital. Parents filled out questionnaires on family background, child’s symptoms, and satisfaction and perceptions of the teaching formats and parenting practices included in the intervention.

**Results:**

Parents liked the parenting program and thought that the parenting practices and the teaching formats were useful and easy to follow. Especially, positive parenting practices were perceived as useful and easy, whereas limit-setting time-out was seen as less useful. Children’s behavioral symptoms had diminished by the end of the program.

**Conclusion:**

Our results suggest that an individually delivered OPPI parenting program is well-liked and perceived as useful in teaching parenting practices and reducing child’s behavioral symptoms in families referred to child psychiatric clinics.

## Introduction

A major reason for referral to child psychiatric outpatient care is behavioral problems (Staller, 2006). Behavioral problems are often highly comorbid with other child psychiatric disorders such as attention-deficit hyperactivity disorder (ADHD), and with family problems. Behavioral problems in childhood are a diverse group of disruptive behaviors that have long-term adverse effects in adolescence and adulthood, including mental health problems, unemployment, and substance use disorders (Fergusson et al., 2015; Scott, 2015). Parenting programs based on social learning theories such as Triple P (Sanders, 1999) Kazdin’s Parent Management Training (Kazdin, 2005) and Incredible Years (Webster-Stratton & Reid, 2017) are known to be effective in reducing children´s behavioral problems. A meta-analysis by Mingebach et al. (2018) reported moderate effect sizes (SMD 0.45, 95% CI 0.35 to 0.55), for such programs. However, engaging parents in programs has proven to be difficult, with up to half of referred parents either not starting the parental program or discontinuing it (Chacko et al., 2016; Kazdin, 1996; McGoron & Ondersma, 2015; Scott & Dadds, 2009). Especially worrisome is that families with complex needs seem not to benefit from these programs (Laas Sigurðardóttir et al., 2024). Hence, there is a critical need to develop more accessible, engaging, and effective treatment programs for child behavioral problems.

Research indicates that barriers to attend parenting programs are typically related to time constraints, difficulties in scheduling appointments, and privacy concerns (Butler et al., 2020; Koerting et al., 2013). However, most research has concerned programs delivered in group format. While group delivery may be beneficial in offering peer support, it may also increase some barriers, such as privacy concerns, dislike of group activities, difficulties following the program (Koerting et al., 2013), and lack of relevance or poor alliance with the therapist, which have been associated with drop-out (Kazdin, 1997; Kazdin et al., 1997) and poorer treatment outcomes (Shirk & Karver, 2003). Individually delivered parenting programs offer a more tailored approach, enabling counselors to address specific family needs and dynamics perceived as important and valued by parents (Butler et al., 2020). Furthermore, they may also allow surpassing of the previously mentioned barriers, thereby potentially enhancing engagement, feasibility, and treatment effectiveness (Heubeck et al., 2016; Lundahl et al., 2006; Reyno & McGrath, 2006). A deeper understanding of how parents perceive parenting programs is necessary as engagement can be enhanced when parents view the program as relevant, useful, and aligned with their needs, which in turn may contribute to improved child outcomes (Chacko et al., 2016). Studies are discrepant on whether group format or individually tailored treatments are more effective (Barlow & Coren, 2018; Lane et al., 2023), and therefore, more research on individually delivered parenting programs is warranted. Furthermore, little attention has been given to the role of counselors in parenting programs, even though they may be vital for parents’ engagement and responsiveness to the treatment (Berkel et al., 2011; Kazdin & Whitley, 2006; Leitão et al., 2023; Lindsey et al., 2014).

The implementation of individually delivered and tailored parenting programs can significantly enhance parental engagement and treatment effectiveness in clinical contexts. Parents within child psychiatric clinical samples frequently feel overwhelmed (Wirehag Nordh et al., 2025) which increases the risk of dropping out or failing to start a parenting program despite a clear need for support (Kazdin & Wassell, 1998). Children are also more likely affected by comorbid mental health issues that may impair the effectiveness of treatment (Reyno & McGrath, 2006). These families are thus recognized as a hard-to-reach group that often needs individually tailored parenting interventions (Barker & Hawes, 2024; Kazdin & Wassell, 1998). Parenting children with behavioral problems is especially challenging and stressful (Barroso et al., 2018). Higher parental stress is associated with the occurrence of negative parenting practices (Crnic et al., 2005). Further, research has indicated that child’s behavioral problems may have a bidirectional association with parental stress, leading to a negative cycle where both contribute to the exacerbation of the other (Kazdin & Wassell, 1998) Especially little is known about parental perceptions and the feasibility of individually delivered parenting programs in child psychiatric settings, where children already show high levels of behavioral problems (Hutchings & Lane, 2005; Kazdin, 1997; Leslie et al., 2005) and parents are often excessively burdened. Previous sparse research mainly from group-delivered parenting programs suggests that parents perceive concrete teaching formats and positive parenting practices as useful and easy to follow (Butler et al., 2020; Leijten et al., 2019). A broader understanding of parents’ perceptions could guide clinicians and program counsellors to target the treatment more effectively and increase relevance in families with high parental distress and children with multiple challenges.

Numerous evidence-based parenting programs have been developed to address children’s disruptive behavior, such as the Incredible Years, Triple P, and Parent Management Training. Meta-analyses show that these programs are effective in improving child behavior and parent–child interaction (Beelmann et al., 2023; Leijten et al., 2022). However, most existing programs are group-based, require extensive therapist training, and involve licensing and cultural adaptation procedures that may limit their implementation in Finnish clinical settings. Moreover, as Barker and Hawes (2024) emphasize, individualized delivery and therapist flexibility are critical when working with families in specialized psychiatric care, where children often present with comorbid conditions and executive function difficulties. These considerations led to the development of the OPPI program, designed to retain the core evidence-based components of parenting interventions (e.g., behavior management, positive reinforcement, relationship enhancement) while allowing for individualized application within Finnish child psychiatric services.

## Aims of the Study

In this study, we introduce and examine the novel OPPI parenting program aiming to understand the aspects that parents of clinically referred children perceive as useful or difficult. A better understanding of parental perceptions could help develop and tailor interventions to be more relevant for families and, consequently, more engaging in the future. Specific research questions encompass two main areas: (1) evaluating participating parents’ overall satisfaction with the program and (2) determining the usefulness and difficulty of teaching formats and parenting practices included in the program. Further, we describe the preliminary effectiveness of the intervention, i.e., whether parents report a change in children’s behavioral symptoms from pre- to post-intervention. We hypothesize that the intervention decreases behavioral symptoms.

## Method

### The Intervention

The manualized parent training program OPPI is an individually delivered intervention designed to reduce child’s behavioral problems by modifying parents’ behavior. The program is developed at Helsinki University Hospital in a research project lead by one of the authors (E.A., project TYH2018203) and funded by Helsinki University Hospital research funds. The research project aimed to develop a Finnish parenting skills training program for patients with conduct problems and evaluate the feasibility and acceptability of the program. The authorship of the program belongs to the research project leader. The OPPI program is based on: (1) knowledge of plasticity of brains during child development (Gibb and Kolb, 2018; Johnson & de Haan, 2015) (2) well-known characteristics of children with behavioral problems (problems with executive functions, self-regulation, language) (Huhdanpää et al., 2019; Teivaanmäki et al., 2020; Xie et al., 2018) and their parents (stress) (Barroso et al., 2018), (3) developmental theories such as coercive interaction theory (Patterson, 1982), cognitive-behavioral (Beck, 1963), social learning (Bandura et al., 1963), attachment, and developmental psychology (Ainsworth, 1969; Bowlby, 1958) approaches, and earlier parenting programs such as Kazdin’s Parent Management Training (Kazdin, 2005) and Incredible Years (Webster-Stratton, 2011). An effort was made to include teaching of important parenting skills in a form that would be acceptable and feasible for families in clinical setting.

**Figure 1.**
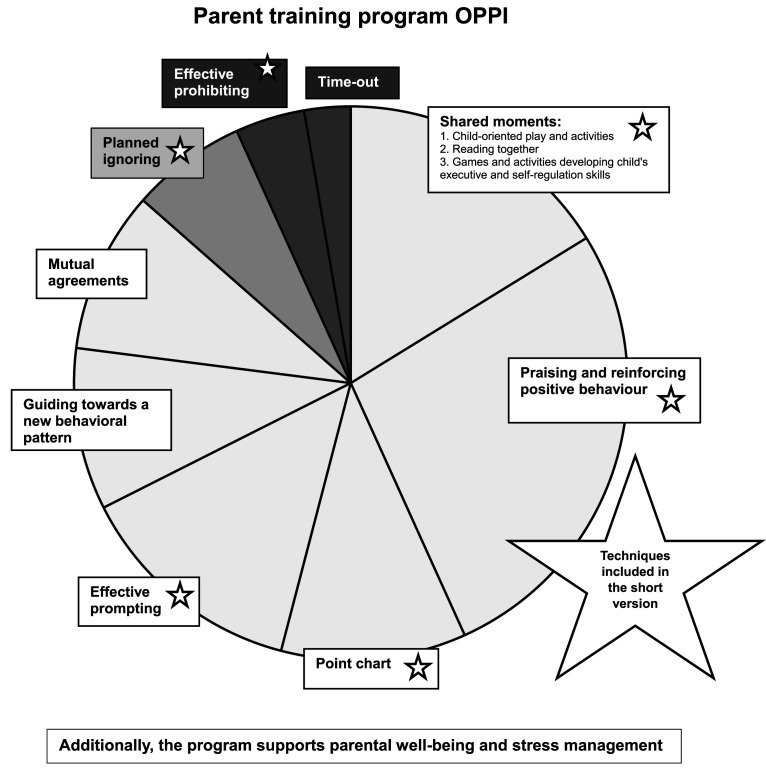
Parenting practices included in the OPPI parenting program. Sector size and color reflect the importance of the specific practice and how much it should be used in daily life. Light color means important positive practice, use often; medium dark and dark means limit setting, use less and deliberately

OPPI is a Finnish word and means learning, highlighting that the program focuses on acquiring knowledge, skills, and understanding related to parenting. It has been developed to meet the needs of child psychiatric patients between 4–13 years and their parents. The main principles of the program include (1) improving the child’s behavior by increasing positive interaction between the child and the parent and (2) providing parents with tools and techniques to reinforce the child’s positive behavior and to reduce and manage negative behavior. The behavior of a child can be influenced by giving attention to it and modifying the factors present before and after the behavior. In addition, the child’s negative behavior decreases when it is ignored or limited consistently, neutrally, and without coercion. The practices and techniques of the parent training program OPPI are shown in Figure 1.

The OPPI program can be performed as a shorter (8 sessions) or longer (12–14 sessions) version based on child age, their estimated symptom severity, parents’ ability to use different skills and to attend sessions, and the number of sessions and skills needed to improve the interaction between child and parent(s). Both parents are invited to participate in the intervention. Separated parents may attend either individually or together. Individual delivery allows for adjustments to better meet the family’s specific needs, although the session topics must follow the manual. For example, the counselor can emphasize particular parenting practices and teaching methods based on the parents’ preferences. Exercises can also be adapted to account for the child’s unique challenges and comorbid symptoms, ensuring the program remains relevant and effective. Each session lasts approximately 1.5 h and can be conducted in person or remotely. During the sessions, parents are taught positive parenting practices through psychoeducational discussions, role-play exercises, written materials and videos in which professional actors play families and demonstrate how parent-child interaction styles affect the child’s behavior. The program includes eight 5- to 14-min videos, first a program and “families” introduction video and then seven videos of parenting skills. In addition, the role of the counselors is to model positive speech and behavior and to maintain a supportive manner of discussions. Throughout the program, the counselors support the parents, for example, by teaching positive internal speech, positive thinking of the future development of the child, and stress reduction skills. Between sessions, parents are assigned to practice the skills with the child and receive encouraging text messages or e-mails from the counselor, being able to reply and ask questions. Although the sessions are primarily for parents, the child is asked to participate in one guidance session (family session). The program includes manuals for both parents and counselors.

OPPI counselors have backgrounds in psychology, social work, occupational therapy, or nursing and are mental health professionals at clinics. They undergo a 2-day training, a booster session, and regular supervision sessions. Training includes pre-assignments and independent study materials. To become an accredited counselor, they must counsel at least three families using the OPPI program and participate in at least six supervision sessions. Supervisors are mental health professionals who have developed the OPPI program, trained the counselors, and provided OPPI interventions at child psychiatric clinics.

The OPPI program has been delivered in child psychiatric clinics at Helsinki University Hospital, Finland since 2019. It is targeted at parents of children with disruptive disorders or symptoms, and participation requires a doctor’s referral. The inclusion criterion is a score of 4 or more on the Strengths and Difficulties Questionnaire’s (SDQ) behavioral difficulties subscale (children under 7 years must have scored 3 points or higher) based on suggested clinical cut-off for conduct subscale (Finnish norms: (Borg et al., 2014) and validation and reliability: (Koskelainen et al., 2000), see for more https://www.sdqinfo.org/). Exclusion criteria comprise severe developmental disorders such as autism spectrum disorders, severely stressful family situations, or ongoing foster care placement processes.

### Participants and Procedure

The data on the OPPI program were collected in naturalistic settings at child psychiatric clinics during program deployment. A total of 97 children were referred to the OPPI parenting program at the outpatient child psychiatry clinics for children in Helsinki University Hospital, between August 2021 and December 2023. The referral was done by doctors based on the intervention inclusion and exclusion criteria. Either both parents of the child or one parent participated in the intervention. All Finnish-speaking parents who attended the intervention were eligible to participate in the study and were asked by the counselor to provide informed consent electronically at the first OPPI session. Of the referred children, 6 did not meet the inclusion criteria, and 19 (19.6%) failed to start or finish the intervention (*n* = 13 unable to participate, *n* = 6 drop-out). The parents of 27 children attended the intervention but did not complete either the pre- or post-questionnaire. The pre-questionnaire included background information, and the post-questionnaire assessed parental satisfaction. Therefore, families who did not complete either questionnaire could not be included in the analyses.

A total of 61 parents of 45 children finished the program, filled out the pre-and post-intervention measures with electronic questionnaires. The diagnoses of these children were not available for the study group. First, the parents filled out the starting questionnaires (baseline information on family and child characteristics and child’s behavioral symptoms). At the end of the intervention, parents reported again the child’s behavioral symptoms and were also asked about their perceptions regarding the program and the counselor. The program was implemented during the COVID-19 pandemic forcing the clinic to organize remote appointment possibilities; therefore, the program could be delivered flexibly, remotely or in-person, according to the family’s needs. Unfortunately, the percentage of remote and in-person appointments was not available from the data. For families there were no costs for attending the program, and they were not paid for participating in this study.

### Measures

#### Sociodemographic Background

The background questionnaire at pre-intervention included information on the children (age, sex, special support at daycare or school, medication), the parents (age, marital status, education, employment, parental perceived stress), and family circumstances (number of siblings, divorce, disagreement about parenting practices).

#### Parental Perceptions of the Program

Parental perceptions of the program, the professional’s delivery of the intervention, and the usefulness and difficulty of teaching formats and program content were evaluated by using a modified Parents Consumer Satisfaction Questionnaire (Forehand & McMahon, 1981) at the end of the intervention. The questionnaire was modified for the current study to better match the intervention. The included version of the PCSQ is provided in Appendix 1. It has 42 items and is rated by using a seven-point Likert scale. Internal consistencies were not calculated for the PCSQ, as we do not treat the questionnaire or its subscales as a sum scale measuring a single underlying concept.

The overall satisfaction subscale consists of six items in which parents evaluated the usefulness and effectiveness of the program for their child’s behavioral problems, whether they would recommend the program to others, and their overall opinion of the program. Parents also evaluated professionals’ teaching and preparation with two items. For each of the items, frequencies and a mean score were calculated.

Treatment format usefulness and difficulty subscales consist of seven items each, in which parents evaluated the usefulness of the teaching methods and how difficult it was to follow them. Items were session content, demonstration of practices by the counselor, practicing skills with professionals, practicing with the child at home, other homework assignments, handouts, and video examples. For each of the variables, a mean score was calculated.

Parenting practices usefulness and difficulty subscales consist of ten items each, in which parents evaluated the usefulness and difficulty of practices taught in the program: shared moments, praising and reinforcing positive behavior, point chart, effective prompting, guiding towards a new behavior pattern, planned ignoring, effective prohibiting, time-out, and the overall group of parenting practices. For each of the variables, a mean score was calculated.

#### Parental Stress

Parental perceived stress was evaluated at the beginning and at the end of the intervention, on a visual analog scale from 0 to 10, where 0 indicates no stress and 10 is the highest level of stress. The causes of the stress were evaluated by eight yes-no items: work/studies, intimate relationship, parenting, home life, social life, financial problems, child symptoms/disorders, or something else. This one-item stress severity scale corresponded well (r = 0.749, *p* < 0.001) with the 10-item validated Perceived Stress Scale, PSS (Cohen et al., 1983) in a Finnish large community sample of parents of school-aged children (*n* = 772) (unpublished).

#### Child Symptoms

Child symptoms were assessed at the beginning and at the end of the intervention, with the Nisonger Child Behavior Rating Form – Typical IQ (NCBRF-TIQ) (Barterian et al., 2017), which is a parent-rated questionnaire to assess problem behavior among children aged 3 to 16 years (Aman et al., 2008). Higher scores indicate more problem behavior, and items are rated on a 4-point frequency/severity scale *(0 = did not occur or was not a problem, 1 = occurred occasionally or was a mild problem, 2 = occurred quite often or was a moderate problem, 3 = occurred a lot or was a severe problem*). Items form six symptom subscales, two of which were used in the current study: Conduct Problems and Oppositional subscales. Further, to calculate Disruptive behavior (*D-total*) the two subscales are combined. Internal consistency of the D-total subscale was excellent at pre-intervention (α = .94) and post-intervention (α = .93). NCBRF-TIQ is a well-established measure of problem behavior, and it has also been validated in children with no intellectual disabilities (Barterian et al., 2017).

### Statistical Methods

For data analysis, SPSS 29 was used. Dependent samples t-tests were applied to assess the differences between child symptoms pre- and post-intervention. McNemar’s test was used to assess change in parents’ stress sources pre- and post-intervention.

## Results

### Baseline Characteristics of the Families

The age range for the 45 children was 4.8–13.8 years (M 9.0, SD 2.4). The majority (84.4%) were boys, 76.7% had special support at daycare or school (information missing for two children), and 71.1% had medication for psychiatric or neuropsychiatric symptoms. Pre-intervention behavioral symptoms are presented in Table 1.

**Table 1.** Child Behavioral Symptoms Assessed by Nisonger Scale at Pre- and Post-Intervention

Nisonger symptoms	Pre-intervention (*n* = 43)			Post-intervention (*n* = 43)			t (df = 42)	*p*-value	Effect size^a^
	Mean	SD	Observed range	Mean	SD	Observed range			
Oppositional	20.47	5.83	7–27	15.28	5.79	4–27	5.451	<.001	0.83
Conduct problem	18.37	9.25	1–41	10.73	6.77	0–31	6.933	<.001	1.10
Disruptive behavior D-total	38.84	13.98	10–68	26.01	11.86	6–58	6.743	<.001	1.03

Disruptive behavior D-total symptom scale is the combination of Oppositional and Conduct symptoms.

^a^Cohen’s d was used to estimate the effect size.

Of the participating parents (*n* = 61), 42 were mothers and 19 were fathers. For 15 children, both parents filled in the questionnaire. Of the parents, 63.2% had higher education (university or university of applied sciences), 26.3% had intermediate vocational education, and 10.5% had no professional training (information missing for four parents). Further, 82.7% were currently employed (information missing for nine parents). Of the parents, 23.3% were divorced or separated (information missing for one parent), and 44.4% reported having disagreements about parenting practices (information missing for seven parents).

### Parental Stress

The mean parental overall stress level at the beginning was 6.7 (SD 2.08). The main causes of the stress were parenting (80.3%), home life (80.3%), child’s symptoms (72.1%), and work (72.1%). Other reported causes of stress were financial problems (47.5%), relationships (41.0%), social life (13.1%), and other (11.5%). The mean stress level at the end of the intervention was 6.6 (SD 2.46). The difference in overall stress between pre- and post-assessment was not significant (*p* = .715). The main causes of the stress at the end were work (72.1%), home life (65.6%), child’s symptoms (63.9%), and parenting (55.7%). Other reported causes of stress were financial problems (45.9%), relationships (32.8%), social life (11.5%), and other (9.8%). Significantly fewer parents reported parenting as a source of stress post-intervention than pre-intervention (*p* = .006). Changes in other sources of stress were not significant.

### Parents’ Overall Satisfaction With the Program

**Figure 2.**
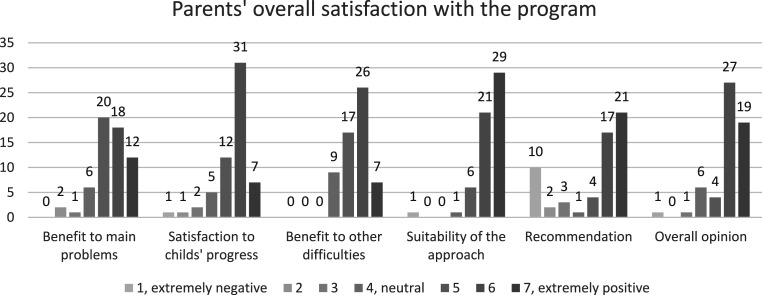
Frequencies of parents reporting satisfaction and benefits of the OPPI parenting program on a scale of 1 to 7 where 1 is extremely negative, 2 negative, 3 somewhat negative, 4 neutral, 5 slightly positive, 6 positive, and 7 extremely positive

Results are shown in Figure 2. Parents’ overall satisfaction with the program, particularly concerning their child and family, was high (M 5.91, SD 1.19). Of the parents, 86.3% (*n* = 50) had positive (5 or higher) overall opinions about the program, and 32.8 % (*n* = 19) reported having extremely positive opinions. Two parents (3.4%) had a negative opinion and six (10.3%) remained neutral.

Parents were satisfied with their child’s progress in general after attending the OPPI parenting program (M 5.49; SD 1.19). Most parents (84.7%, *n* = 50) reported that there was an improvement at the end of the program in the problems for which they had attended the OPPI intervention, three reported that their child’s symptoms were at least slightly worse, and six remained neutral. Most parents (96.5%, *n* = 56) reported that using this type of intervention for child behavioral problems at home is appropriate.

Of the parents, 42 would recommend the intervention for someone close to them and 15 would not recommend the intervention; of these parents, 10 would strongly not recommend the intervention. Of these 15 parents that would not recommend the program, 13 reported, curiously, that using this type of intervention for child behavioral problems at home is appropriate and 11 reported improvements in the problems for which they had attended the intervention.

### Parents’ Satisfaction With the Counselor

**Figure 3.**
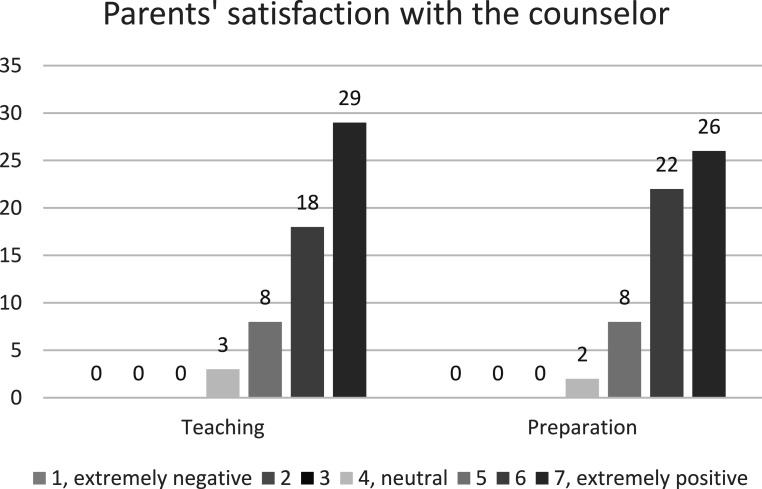
Frequencies of parents’ satisfaction with the counselor’s teaching and preparation on a scale of 1 to 7, where 1 is extremely negative, 2 negative, 3 somewhat negative, 4 neutral, 5 slightly positive, 6 positive, and 7 extremely positive

Parents deemed the information and guidance given by the counselors to be of high quality (M 6.26, SD 0.89) and reported that the counselor’s preparation for meetings and teaching were at least high quality or excellent (82.8% *n* = 48 and 81.0%, *n* = 47, respectively). None of the participating parents reported negative feedback about the counselors. Results are shown in Figure 3.

### Parental Perceptions of the Teaching Format

**Figure 4.**
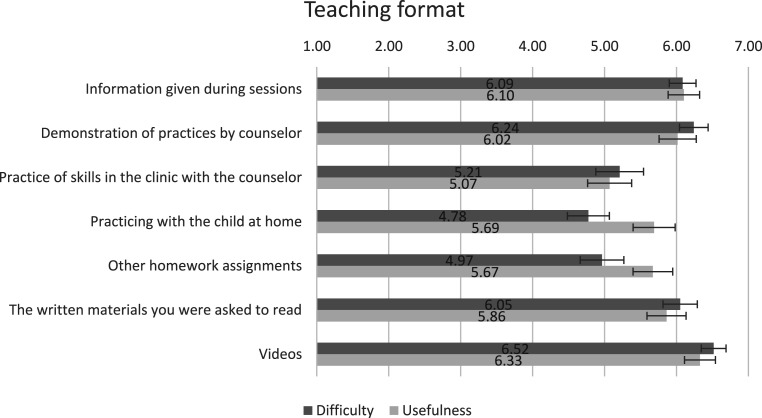
Means and 95% confidence intervals of the usefulness and the difficulty of the teaching formats. Usefulness of the teaching format on a scale of 1 to 7, where 1 is extremely not useful, 2 not useful, 3 somewhat not useful, 4 neutral, 5 somewhat useful, 6 useful, and 7 extremely useful. Difficulty of the teaching format on a scale of 1 to 7, where 1 is extremely difficult, 2 difficult, 3 somewhat difficult, 4 neutral, 5 somewhat easy, 6 easy, and 7 extremely easy

Parental perceptions of the usefulness and the difficulty of the teaching format used in the OPPI parenting program are shown in Figure 4. Parents perceived videos, the information given during sessions, and the demonstration of parenting practices by the counselor to be the most useful teaching methods (mean ˃6 and *n* = 53, *n* = 55, *n* = 56 of the parents, respectively, rated as useful). Among specific teaching methods, practicing at home was perceived as the least easy (M 4.78, SD 1.11) but useful (M 5.69, SD 1.11).

### Parental Perceptions of Parenting Practices Included in the Intervention

**Figure 5.**
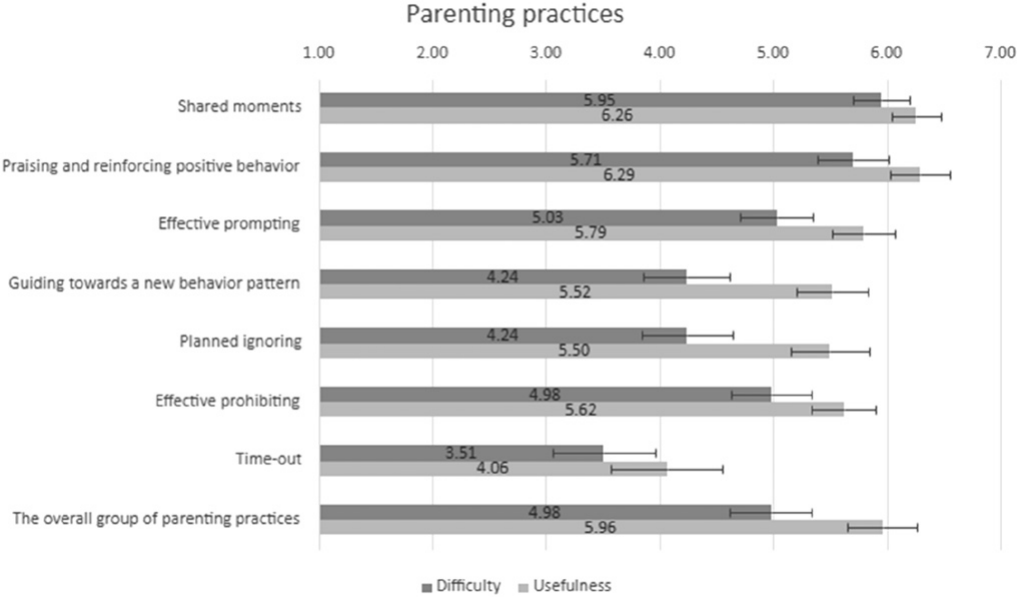
Means and 95% confidence intervals of the usefulness and the difficulty of parenting practices. Usefulness of the parenting practice on a scale of 1 to 7, where 1 is extremely not useful, 2 not useful, 3 somewhat not useful, 4 neutral, 5 somewhat useful, 6 useful, and 7 extremely useful. Difficulty of the parenting practice on a scale of 1 to 7, where 1 is extremely difficult, 2 difficult, 3 somewhat difficult, 4 neutral, 5 somewhat easy, 6 easy, and 7 extremely easy

Parental perceptions of the usefulness and the difficulty of parenting practices included in the OPPI parenting program are shown in Figure 5. Overall, parents perceived the program’s parenting practices as useful (M 5.5–6.29), except for time-out. Among specific practices, praising and reinforcing positive behavior and shared moments had the highest mean scores (M 6.29, SD 1.01 and M 6.26, SD 0.83, respectively) and were perceived as useful by most parents (*n* = 56). Time-out was perceived as the least useful (M 4.06, SD 1.69) and the most difficult (M 3.51, SD 1.45) of the practices. Guiding towards a new behavior pattern and planned ignoring were perceived as the next most difficult (M 4.24, SD 1.41 and mean 4.24, SD 1.53, respectively), albeit useful (M 5.5, SD 1.14 and M 5.5, SD 1.33, respectively), parenting practices.

### Child Symptoms

Child symptom ratings were available for 43 children and when both parents’ evaluations were available, only the first response was included. Results are shown in Table 1.

Parents reported that their child’s behavioral problems decreased from pre- to post-intervention (mean change for D-total scale = 12.83 points, *p* < .001), and both oppositional and conduct symptoms decreased significantly from pre-intervention to the program’s end (both *p*-values <.001). Cohen´s d was used to estimate effect size, effect sizes were statistically large (varying from d = 0.83 to d = 1.10).

## Discussion

The study introduced the novel parenting program OPPI, developed to reduce child behavioral problems in child psychiatric settings. We investigated parental perceptions of the program´s content and teaching formats, and the change in child behavioral symptoms.

Parents were satisfied with the OPPI parenting program in general, they perceived to benefit from it and reported that the program mitigated the major problems for which they had attended. Most parents were extremely satisfied with the trained professionals and perceived the program format and content to be useful. Parent-reported child disruptive behavior symptoms decreased significantly during the program.

The satisfaction with and the benefit from the OPPI parenting program are mostly in line with the previous literature focusing mainly on group-delivered parenting programs (e.g., as Triple P (Sanders, 1999) and Incredible Years (Webster-Stratton & Reid, 2017)) or child’s behavioral problems and finding the programs to be accepted, recommended, and effective in earlier studied populations (Barlow & Coren, 2018; Comer et al., 2013; De Graaf et al., 2008; Furlong et al., 2012; Lane et al., 2023; Leijten et al., 2019; Skinner et al., 2024). However, earlier studies are rare in child psychiatric populations, and individually delivered programs have mainly been investigated in different subgroups (Lane et al., 2023). Our findings align with recent meta-analyses linking the effectiveness of parenting programs more on core components than on the specific program model (Leijten et al., 2022; Tehrani et al., 2024). This supports the rationale for developing OPPI as an individualized, manualized intervention that integrates these empirically supported techniques into a flexible format suitable for specialized clinical practice where children have complex needs and might not benefit from group-delivered programs (Laas Sigurðardóttir et al., 2024). In contrast to widely disseminated group-based programs, OPPI was designed for one-on-one implementation, which may improve accessibility and engagement (Barker & Hawes, 2024; Beelmann et al., 2023). The program has a clear structure and content, but its length can be tailored based on the child’s characteristics and the family’s situation. Individual sessions allow for adjustments to meet the family’s specific needs better, although the session topics must follow the manual.

Unexpectedly, the recommendation rate of 72.4% was lower than in previous reports (e.g., recommendation rates of 87.5%–100% (Axberg & Broberg, 2012; Karjalainen et al., 2021). Yet, the recommendation rate of over 70% in child psychiatric sample can be considered rather high as this type of intervention is inevitably demanding and time-consuming for stressed parents. In our sample parents perceived stress about child symptoms, which might affect their willingness to recommend program to others. Parents’ stress levels remained high throughout the intervention possibly reflecting parents’ stress and demands overall, beyond parenting practices.

### Program Teaching Formats and Parenting Practices

Parents perceived the teaching format and program content to be useful, which is generally consistent with previous studies of similar programs (e.g., Kazdin PMT, Triple P, Incredible Years) (Barlow & Coren, 2018; Butler et al., 2020; Kane et al., 2007). Practical teaching methods, videos, information given during sessions and demonstration of skills, were perceived by parents as both the easiest and the most useful. This may reflect the need to use simple and concrete teaching methods when working with parents. Since parents are often under considerable stress, practical and straightforward instructional approaches may make it easier for them to adopt new parenting strategies during everyday life. In our study, practicing at home was considered harder than many of the other formats. This is also supported in earlier studies (Butler et al., 2020). Practicing at home was nonetheless mostly rated as beneficial. Previous reports have noted difficulties in finding time for home practice and home practice not being enjoyable (Butler et al., 2020), which is consistent with feelings of hardship despite being useful. Regarding the finding that home practice was perceived as the most challenging teaching method, might be understandable as adopting a new behavioral model is difficult in general. Counselors contacted parents between sessions and parents were able to ask for support. However, in this intervention, practice at home was conducted without the counselor’s immediate presence. This may be overly demanding for families experiencing high stress or when a child is having severe symptoms. These families might need extra support to transfer these parenting skills and new behavioral models to everyday life. For example, home visits could be added for these families (McTaggart & Sanders, 2007; Strauss et al., 2012).

In accord with earlier research, parents in our study perceived positive parenting practices, such as providing positive attention and positive reinforcement, to be the most beneficial practices (Butler et al., 2020; Leijten et al., 2019). However, the “shared moments” practice was perceived almost as useful and even easier to apply than positive reinforcement. In this program, the shared moments practice included child-led play or other activities and dialogic reading. Also, games and activities promoting the development of executive and self-regulation skills and parent-child interaction were included. These everyday practices were included as many children with behavioral problems have deficits in language development and executive function (Huhdanpää et al., 2019; Teivaanmäki et al., 2020; Xie et al., 2018).

### Child’s Disruptive Symptoms

Parents reported that children’s disruptive behavior, both oppositional and conduct problems, decreased significantly from the start to the conclusion. As this study is preliminary with respect to disruptive symptom release and we did not have a control group, no direct conclusions about the effectiveness of the program for symptoms can be drawn. However, as the mean disruptive symptoms total score decreased by 13 points and the effect sizes (d = 0.83–1.10) were higher than previously reported in similar populations (previously reported mean effect size d = 0.45, Mingebach et al., 2018), the parents clearly perceived the symptoms to decrease. The general perceived stress level of parents did not change from the pre- to post-intervention, and this would suggest that the program effects were not mediated by change in parental stress.

### Participation in the OPPI Program at Clinics

Of the 97 families referred to the OPPI program at clinics, 25 (25.8%) either could not start or finish the intervention. Of these 25 families, 6 did not meet the inclusion/exclusion criteria, 13 were unable to participate, and 6 families dropped out during the program. Of the 78 families that started the program, only 7.7% dropped out, which is a smaller attrition rate than often reported in parenting programs (Butler & Titus, 2015; Chacko et al., 2016). One contributing factor for low attrition rate might be that the program is individually delivered, and can be tailored based on the child’s age, estimated symptom severity, parents’ ability to use different skills and to attend sessions, and the number of sessions needed to improve the interaction between child and parent(s). Tailoring might lower the barriers for families to engage in the intervention by enabling the adjustment of the focus, content and counseling formats based on each family’s individual needs (Butler et al., 2020; Butler & Titus, 2015).

### Strengths and Limitations

Strengths of this study include the development of a new, individually delivered parenting program and the unique study population, as it is important to investigate the acceptability and effectiveness of parenting programs in the specialized care child psychiatry population. Additionally, we offer a comprehensive analysis of the program’s formats and contents. The results support clinicians in identifying key areas to prioritize during treatment and in using the most useful and easy training methods as part of other treatment modalities. Some drawbacks of our study must also be addressed, and further investigation is needed on these matters. Qualitative data on parents’ perspectives would have improved the understanding of the program’s benefits and challenges and given richer insights into parents’ views. In addition, we did not collect information on whether a co-parent attended all, some, or none of the intervention appointments, nor did we record the percentage of meetings that were held remotely versus in person. The moderate sample size does not allow for more elaborate statistical analyses in subgroups of the sample. Parents in our study were mostly rather highly educated, which might not be representative of the program’s clinical target population and limits the generalizability of the results to all patients. Limitations also include the lack of a control group and the use of only the parent report on the child’s symptoms. Hence, the results of the effectiveness of the intervention for behavioral symptoms of the child are merely preliminary. It is also possible that parents’ opinions of the program might reflect the desire to please the counselor even though the reports were anonymous. Including multiple informants, such as clinicians and teachers, would be an important addition to future studies of the program.

### Clinical and Practical Implications

Based on our study, we believe that families attending specialized care can be engaged in an individualized and tailored parenting program. Our results on the usefulness and the difficulty of the program teaching format and content may help to develop and tailor interventions to be more relevant for families. Most children with behavioral problems in clinical settings also have other symptoms or comorbid disorders and family stress. Parenting programs in clinical settings should be considered as part of the total treatment plan, as the child may also need other treatment. Offering a choice of interventions with varying levels of intensity and flexibility tailored to the specific needs of families is likely to be beneficial. Yet, it should be further studied if individual delivery increases parents’ engagement in child psychiatric care. As the OPPI program was delivered by counselors working at the child psychiatric clinics, co-working with other personnel was possible. In many cases this helped the counselors to follow the OPPI schedule as other needs were taken care of by other professionals included in the child’s treatment team. As practicing at home is considered an important part of parenting programs and difficulties in this might lead to a decrease in intervention effectiveness. Our results suggest that parents need additional support to effectively transfer the parenting practices learned during the intervention to the home setting.

## Conclusions

The preliminary results of the individually delivered OPPI parenting program are promising and suggest that it is both acceptable and potentially effective for families in child psychiatric care. In future, randomized controlled studies on the effectiveness are needed. The results highlight the need for flexible and tailored parenting interventions as part of the total treatment plan in clinical settings. It is noteworthy that a surprisingly large proportion of parents who expressed high levels of satisfaction with the OPPI program would not recommend it to others, a finding that requires further investigation. Future studies with larger samples and control groups are warranted to assess the engagement, efficacy, feasibility, and long-term effects of the OPPI program. The present study provides valuable information on the usefulness and the difficulty of the program teaching format and content that may help to develop and tailor interventions to be more relevant for families.

## Supplemental Material

Supplemental Material - Individually Delivered Parenting Program OPPI: Promising Results in Parental Perceptions and Children’s Behavioral Symptoms in Clinical Settings Supplemental Material for Individually Delivered Parenting Program OPPI: Promising Results in Parental Perceptions and Children’s Behavioral Symptoms in Clinical Settings by Assi Peltonen, Vilja Seppälä, Heidi Backman, Taru Saarelainen, Tiia Kuha, Marjo Flykt, Eeva T. Aronen in Clinical Child Psychology and Psychiatry

## Data Availability

Patient data are available only for the study group.*
