# Supplementary material for: Individually Delivered Parenting Program OPPI: Promising Results in Parental Perceptions and Children’s Behavioral Symptoms in Clinical Settings
Source: Clin Child Psychol Psychiatry. 2025 Dec 19;31(2):473–93. doi: 10.1177/13591045251407367 (PMC12992632; doi:10.1177/13591045251407367)
Supplement: Supplemental Material - Individually Delivered Parenting Program OPPI: Promising Results in Parental Perceptions and Children’s Behavioral Symptoms in Clinical Settings [file sj-pdf-1-ccp-10.1177_13591045251407367.pdf]

Appendix 1. Parents' consumer satisfaction questionnaire.
Overall

Please circle the response that best expresses how you honestly feel.

	1	2	3	4	5	6	7
1. The major problems that originally prompted me to treatment for my child is (are) at this point?	considerably worse	worse	slightly worse	the same	slightly improved	improved	greatly improved
2. My feelings at this point about my child´s progress are that I am	very dissatisfied	dissatisfied	slightly dissatisfied	neutral	slightly satisfied	satisfied	very satisfied
3. To what degree has the treatment program helped with other general personal or family problems not directly related to your child?	hindered much more than helped	hindered	slightly hindered	neither helped or hindered	helped slightly	helped	helped very much
4. I feel the approach to treating my child´s behavioural problems in the home by using this type of parent training program is	very inappropriate	inappropriat e	slightly inappropri ate	neutral	slightly appropriate	appropriate	very appropriate
5. Would you recommend this program to a friend or relative	strongly not recommende d	not recommend ed	slightly not recommen ded	neutral	slightly recommende d	recommend ed	strongly recommende d
6. My overall feeling about the treatment program for my child and	very negative	negative	slightly negative	neutral	slightly positive	positive	very positive

family is

Therapist(s)

In this section we'd like to get your ideas about your therapist(s). Please circle the response to each questions that best expresses how you honestly feel.

	1	2	3	4	5	6	7
I feel that the therapist's teaching was	poor	fair	slightly below average	average	slightly above average	high	superior
The therapist's preparation was	poor	fair	slightly below average	average	slightly above average	high	superior

Teaching format

In this section, we´d like to get your ideas of how difficult each of the following types of teaching has been for you to follow. Please circle the response that most clearly describes your opinion.

	1	2	3	4	5	6	7
1. Lecture information	extremel y difficult	difficul t	somewha t difficult	neutra l	somewha t easy	easy	extremel y easy
2. Demonstratio n of skills by the therapist	extremel y difficult	difficul t	somewha t difficult	neutra l	somewha t easy	easy	extremel y easy
3. Practice of skills in the clinic with the therapist	extremel y difficult	difficul t	somewha t difficult	neutra l	somewha t easy	easy	extremel y easy
4. Practice of skills in the clinic with your child	extremel y difficult	difficul t	somewha t difficult	neutra l	somewha t easy	easy	extremel y easy
5. Other homework assignments	extremel y difficult	difficul t	somewha t difficult	neutra l	somewha t easy	easy	extremel y easy
6. The written materials you were asked to read	extremel y difficult	difficul t	somewha t difficult	neutra l	somewha t easy	easy	extremel y easy
7. Video examples	extremel y difficult	difficul t	somewha t difficult	neutra l	somewha t easy	easy	extremel y easy

In this section, we´d like to get your ideas of how useful each of the following types of teaching is for you now. Please circle the response that most clearly describes your opinion.

	1	2	3	4	5	6	7
1. Lecture information	extremel y not useful	not useful	somewha t not useful	neutra l	somewha t useful	usefu l	extremel y useful
2. Demonstratio n of skills by the therapist	extremel y not useful	not useful	somewha t not useful	neutra l	somewha t useful	usefu l	extremel y useful
3. Practice of skills in the clinic with the therapist	extremel y not useful	not useful	somewha t not useful	neutra l	somewha t useful	usefu l	extremel y useful

4. Practice of skills in the clinic with your child	extremely not useful	not useful	somewhat not useful	neutral	somewhat useful	useful	extremely useful
5. Other homework assignments	extremely not useful	not useful	somewhat not useful	neutral	somewhat useful	useful	extremely useful
6. The written materials you were asked to read	extremely not useful	not useful	somewhat not useful	neutral	somewhat useful	useful	extremely useful
7. Video examples	extremely not useful	not useful	somewhat not useful	neutral	somewhat useful	useful	extremely useful

Specific parenting techniques

In this section, we´d like to get your idea of how difficult it usually is to do each of the following techniques now. Please circle the response that most closely describes how diffult the technique is to do.

	1	2	3	4	5	6	7
1. Shared moments	extremely difficult	difficult	somewhat difficult	neutral	somewhat easy	easy	extremely easy
2. Praising and reinforcing positive behavior	extremely difficult	difficult	somewhat difficult	neutral	somewhat easy	easy	extremely easy
3. Point chart	extremely difficult	difficult	somewhat difficult	neutral	somewhat easy	easy	extremely easy
4. Planned ignoring	extremely difficult	difficult	somewhat difficult	neutral	somewhat easy	easy	extremely easy
5. Effective prompting	extremely difficult	difficult	somewhat difficult	neutral	somewhat easy	easy	extremely easy
6. Time-out	extremely difficult	difficult	somewhat difficult	neutral	somewhat easy	easy	extremely easy
7. Promoting new behavioral patterns	extremely difficult	difficult	somewhat difficult	neutral	somewhat easy	easy	extremely easy
8. Effective prohibiting	extremely difficult	difficult	somewhat difficult	neutral	somewhat easy	easy	extremely easy
9. Mutual agreements	extremely difficult	difficult	somewhat difficult	neutral	somewhat easy	easy	extremely easy
10. The overall group of techniques	extremely difficult	difficult	somewhat difficult	neutral	somewhat easy	easy	extremely easy

In this section, we´d like to get your idea of how useful each of the following techniques is to you in improving your interaction with child and decreasing his or her "not ok" behavior now. Please circle the response that most closely describes the usefulness of the techniques.

	1	2	3	4	5	6	7
1. Shared moments	extremely not useful	not useful	somewhat not useful	neutral	somewhat useful	useful	extremely useful
2. Praising and reinforcing positive behavior	extremely not useful	not useful	somewhat not useful	neutral	somewhat useful	useful	extremely useful
3. Point chart	extremely not useful	not useful	somewhat not useful	neutral	somewhat useful	useful	extremely useful
4. Planned ignoring	extremely not useful	not useful	somewhat not useful	neutral	somewhat useful	useful	extremely useful
5. Effective prompting	extremely not useful	not useful	somewhat not useful	neutral	somewhat useful	useful	extremely useful
6. Time-out	extremely not useful	not useful	somewhat not useful	neutral	somewhat useful	useful	extremely useful
7. Promoting new behavioral patterns	extremely not useful	not useful	somewhat not useful	neutral	somewhat useful	useful	extremely useful
8. Effective prohibiting	extremely not useful	not useful	somewhat not useful	neutral	somewhat useful	useful	extremely useful
9. Mutual agreements	extremely not useful	not useful	somewhat not useful	neutral	somewhat useful	useful	extremely useful
10. The overall group of techniques	extremely not useful	not useful	somewhat not useful	neutral	somewhat useful	useful	extremely useful
